# Antibacterial and antioxidant activity of *Juniperus thurifera* L. leaf extracts growing in East of Algeria

**DOI:** 10.14202/vetworld.2018.373-378

**Published:** 2018-03-27

**Authors:** Merradi Manel, Heleili Nouzha, Mekari Rim, Mekkaoui Imane, Aouachria Sana, Oucheriah Yasmine, Ayachi Ammar

**Affiliations:** 1 Department of Microbiology-Biochemistry, Faculty of Natural and Life Sciences, Batna2 University, Batna, Algeria; 2 Department of Veterinary Sciences, Institute of Veterinary Sciences and Agronomy Sciences, University of Batna, Batna, Algeria

**Keywords:** antibacterial activity, antioxidant activity, extraction, *Juniperus thurifera* L, trapping test of 1,1-diphenyl-2-picrylhydrazyl radical

## Abstract

**Aim:**

This work aimed to evaluate the biological activity of the leaf extracts of *Juniperus thurifera* L., which is an Algerian endemic tree that belongs to the family of Cupressaceae.

**Materials and Methods:**

The plant leaves were extracted in solvents of increasing polarity to obtain different extracts such as methanol, petroleum ether, chloroform, ethyl acetate, and aqueous extracts (MeE, PEE, ChlE, EtAE, and AqE). The antioxidant activity of four extracts (MeE, ChlE, EtAE, and AqE) was assessed by trapping test of 1,1-diphenyl-2-picrylhydrazyl (DPPH) radical. The evaluation of antibacterial activity of MeE, ChlE, EtAE, and PEE was done using the disk diffusion method on solid agar.

**Results:**

The three extracts of EtAE, AqE, and MeE showed high antiradical activity toward the DPPH radical (IC_50_=29.348 µg/mL, 37.538 µg/mL, and 52.573 µg/mL, respectively), while the lowest radical scavenging activity was expressed by the ChlE (IC_50_=70.096 µg/mL). These extracts were active only toward the Gram-positive bacteria (*Staphylococcus aureus* ATCC and methicillin-resistant *S. aureus*) at different concentrations, and the highest activity was obtained with the ChlE with an inhibition diameter of 14 mm at the concentration of 1 g/mL. No inhibition was detected for all of these extracts against the Gram-negative tested strains (*Escherichia coli* ATCC, *Pseudomonas aeruginosa* ATCC, and *Enterobacter cloacae* (extended spectrum β-lactamase).

**Conclusion:**

From this study, on the one hand, it was concluded that *J. thurifera* L. leaves extracts exhibited a very intense antioxidant potential toward the DPPH radical, and on the other hand, the antibacterial activity showed an action spectrum exclusively toward the Gram-positive bacteria.

## Introduction

Thousands of years ago, using plant was always a folklore trailed by traditional healers, yet the World Health Organization reports that about 65-80% of the world’s population in developing countries, due to poverty and lack of access to modern medicine, rely heavily on traditional medicinal plants for their primary health care [1,2].Plant extracts are known for their fragrance and flavor [3] and also provide a wide variety of secondary metabolites as volatile oils, polyphenols tannins, alkaloids, flavonoids, terpenoids, and glycosides [4] which have been found *in vitro* to have antifungal, antibacterial, and antiprotozoal activities [5,6]. These natural molecules can be used against microorganisms instead of synthetic chemicals (antibiotics) that cause allergic reaction and immunity suppression as they are less damaging to the human health because they are generally few toxic and they do not have side effects [7] and to make an end for the fail in antibiotic therapy because of the emergence of highly drug-resistant bacteria such as methicillin-resistant *Staphylococcus aureus* (MRSA) and extended spectrum β-lactamase (ESBL)-producing Enterobacteriaceae [8].

Algeria is one of the African countries with a diverse flora, where numerous species are believed to possess curative properties, presenting diverse interests and constitutes an axis of scientific research more particularly in the field of natural substances [9]. The genus *Juniperus* is an important component of arid and semi-arid ecosystems throughout the Northern Hemisphere, which is present in the South of the Mediterranean in the Moroccan Atlas and the Aures of Algeria and in the North of the Mediterranean in Spain, France, and Italy. One of the major genera of Cupressaceae family consisting of approximately 70 species variables in size and shape, from tall trees to columnar or low spreading shrubs [10], is considered as an important medicinal plant largely used in traditional medicine. *Juniperus thurifera* L. plays a special role in the western basin of the Mediterranean and traditionally used for curing different disorders and pathological conditions.

The present study aimed to evaluate the antibacterial and the antioxidant activities of the extracts obtained from the leaves of *J. thurifera* L. growing in the Aures’ Mountains, Eastern Algeria.

## Materials and Methods

### Ethical approval

No animal was used in this study. Hence, ethical approval was not needed.

### Plant material

The plant material consists of *J. thurifera* L. dry leaves. The selected species were collected from its natural habitat. The leaves were harvested from Zana region (Theniet El Abed), Batna, Algeria, on March 13, 2016. After harvesting, the leaves were cleaned and dried at 45°C. The dried plant material was subsequently reduced to powder. The resulting powder was stored away from air, moisture, and light.

## Extraction

The extraction was carried out by subjecting 50 g of dry leaves powder at maceration in methanol (500 mL) in an opaque container, with a manual agitation to ensure that all the surface of the powder was impregnated in solvent and to accelerate the process of extraction [11]. The mixture was filtered through muslin cloth, cotton, and in the end with the folded filter paper successively after a 3 days’ incubation period at room temperature, and the same process was repeated twice. The three filtrates recovered were then evaporated in a Rotavapor to obtain a sunk green extract, which was regarded as being the crude methanol extract (MeE) of leaves. This extract was dried and weighed to determine the yield of extraction. Then, this extract was fractionated using a series of increasingly polar solvents, and thus, three other extracts were obtained: One from petroleum ether (PEE), another from chloroform (ChlE), and a last one from ethyl acetate (EtAE). The resulting raffinate represented the residual aqueous extract (AqE).

### Evaluation of antioxidant activity

The antioxidant activity was evaluated using the scavenging method with modifications where 1,1-diphenyl-2-picrylhydrazyl (DPPH) was used as a relatively stable free radical [12]. A DPPH solution was prepared by dissolving 4 mg of DPPH in 100 mL of methanol. Concentrations of extracts solutions were prepared, and then, we added 50 µL of each concentration to 1250 µL of the DPPH solution, after incubation for 30 min in the dark and at ambient temperature, the absorbances are measured at 517 nm with the corresponding blank. The results were expressed by the mean of three separate measurements ± standard deviation.

The IC_50_ parameter is defined as the effective concentration of the substrate that can trap 50% of the total DPPH. The results expressed in IC_50_ are calculated from the scavenging percentage variation curves depending on the concentration of each extract. The scavenging power is expressed in % and determined by applying the formula:

DPPH free radical scavenging (%) = [(absorbance of control – absorbance of sample)/absorbance of control] ×100

### Evaluation of antibacterial activity

#### Bacterial strains

In total, five bacteria were used for the antimicrobial screening: two were Gram-Positive (*S. aureus* ATCC (25923) and *S. aureus* MRSA) and three Gram-negative (*Pseudomonas aeruginosa* ATCC (27853), *Escherichia coli* ATCC (25922) and *Enterobacter cloacae* (ESBL)).

The Bacterial strains were clinical isolates provided from the Microbiology laboratory, Faculty of Medicine, Batna, Algeria.

### The evaluation method

The dried extracts of *J. thurifera* L. were dissolved in methanol to a final concentration of 100 mg/mL, 250 mg/mL, 500 mg/mL, and 1 g/mL (the EtAE was tested for one concentration 100 mg/mL since its amount was not enough). Antibacterial tests were then carried out by disk diffusion method. The disks are soaked with 10 µL of each extract.

The bacterial strains are seeded on nutrient agar and incubated at 37°C for 24 h, to optimize their growth. After growth, a suspension is prepared with diluted distilled water and adjusted to a concentration of 0.5 Mcf [13]. Bacterial suspensions were streaked over the surface of Mueller-Hinton agar using a sterile cotton swab to ensure uniform inoculation. Then, disks impregnated with 10 µL of extracts are gently placed on the surface of the inoculated agar. Two antibiotics (cefoxitin for Gram-negative bacteria and gentamicin for Gram-positive bacteria) were used as a positive control, and negative control was a disk impregnated with methanol. After incubation for 24 h at 37°C, inhibition zone diameters were measured and documented. The experiment was carried out in duplicate.

## Results and Discussion

### Extraction yield

The MeE of leaves of *J. thurifera* L. was obtained by maceration method with methanol. Then, it was fractionated successively with the solvent of increasing polarity (PEE, ChlE, and EtAE). The percentage of extraction yield was listed as follows: ChlE represents the highest yield (8.8%) relative to the total weight of the dry leaves, and it seems to be in agreement with the extraction yield of the *Juniperus excelsa* leaves [14]; PEE (2.2%); EtAE; and AqE with the same rate (1.2%).

### Evaluation of antioxidant activity

**Figure-1:**
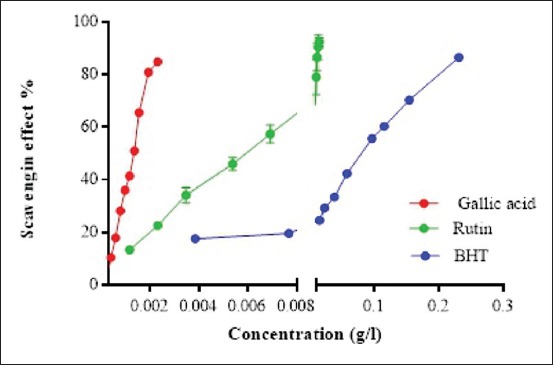
Reference antioxidant antiradical activity. Values are the mean ± SD (n=3).

**Figure-2:**
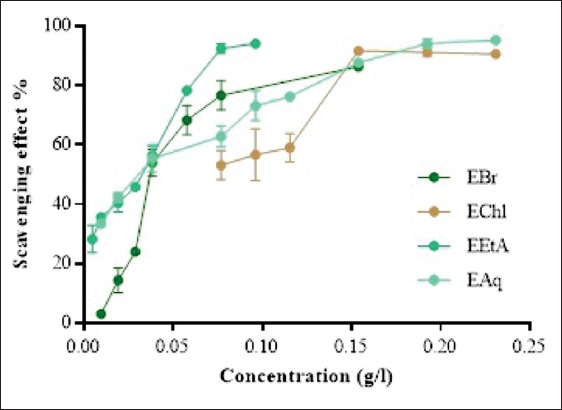
*Juniperus thurifera* L. leaves extracts’ antiradical activity. Values are the mean ± SD (n=3). EBr=Methanolic extract; EChl=Chloroform extract; EEtA=Ethy acetate exctract; EAq=Aqueous extract

Free radical scavenging activity of various extracts of *J. thurifera* L. was tested using DPPH (Figures-1 and 2). The four extracts were able to decolorize the stable, purple-colored radical DPPH into yellow-colored DPPH-H and appear to be concentration dependent. The antioxidant activity of *Juniperus foetidissima* Willd. extract exhibited a good concentration dependent while plant oil was not concentration dependent [15].

During our investigation, it was noted that the studied plant has a good antioxidant activity. The results obtained are in agreement with those announced by several researches [16,17]. It was demonstrated that *Juniperus communis* had the lowest antioxidant activity compared with *Olea europaea* (Olive leaf), *Liquidambar orientalis* (Turkish sweetgum), and *Ziziphus jujuba* (Lotus) probably because they choose another technic than the DPPH method [14].

**Figure-3:**
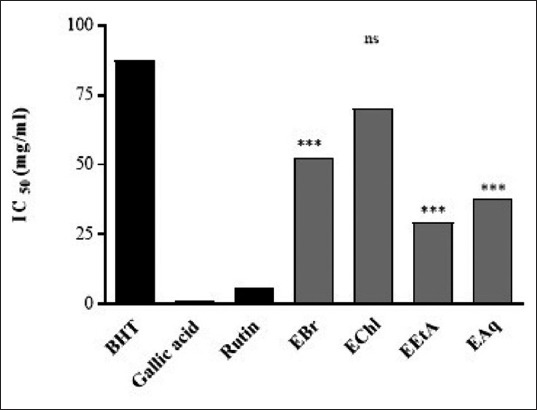
IC_50_ of the antiradical activity of *Juniperus thurifera* leaf extracts and reference antioxidants toward 1,1-diphenyl-2-picrylhydrazyl radical. Values represent mean ± SD (n=3). Values are compared to the BHT. Ns=Not significant, ***p<0.001. EBr=Methanolic extract, EChl=Chloroform extract; EEtA=Ethy acetate exctract; EAq=Aqueous extract

IC_50_ values of each extract are presented in Figure-3. The obtained results allowed us to classify the extracts and standards according to their antiradical capacity as follows: Gallic acid< rutin< EtAE< AqE< MeE< ChlE< BHT. EtAE represents the most active extract (IC_50_=29.348 μg/mL) followed by the AqE with IC_50_=37.538 µg/mL and the crude extract with an IC_50_=52.573 µg/mL.Weli *et al*. [15] demonstrated that EtAE showed less antioxidant activity from *J. excels* leaves, followed by the AqE with IC_50_=37.538 µg/mL and the MeE with an IC_50_ = 52.573 µg/mL. On the other hand, the lowest antiradical activity was expressed by the ChlE that was 2-3 times less active than the EtAE (IC_50_ = 70.096 µg/mL) [14].

### Evaluation of antibacterial activity

The antibacterial activity of *J. thurifera* L. extracts against examined bacteria was quantitatively assessed by the presence or absence of inhibition zones and zone diameters as shown in Table-1. Values are an average of two repeats.

**Table-1:** Inhibition zone diameters of *J. thurifera* L. leaf extracts against bacterial growth.

Strain	Extract											

	MeE				EtAE	ChlE				PEE		
			
	100 mg	250 mg	500 mg	1 g	100 mg	100 mg	250 mg	500 mg	1 g	100 mg	250 mg	500 mg
*S. aureus* ATCC	-	(+) 9	(+) 11	(+) 12	-	-	(+) 9	(+) 11	(+) 11	-	(+) 10	(+) 11
*P. aeruginosa* ATCC	-	-	-	-	-	-	-	-	-	-	-	-
*E. coli* ATCC	-	-	-	-	-	-	-	-	-	-	-	-
*E. cloacae* (ESBL)	-	-	-	-	-	-	-	-	-	-	-	-
*S. aureus* (MRSA)	-	(+) 10	(+) 11	(+) 13	-	-	(+) 10	(+) 12	(+) 14	-	(+) 9	(+) 9

No inhibition was observed with the negative control (methanol), which proves that the solvent could not act as an antibacterial agent. *S. aureus=Staphylococcus aureus, P. aeruginosa=Pseudomonas aeruginosa, E. coli=Escherichia coli, E. cloacae=Enterobacter cloacae, ESBL=*extended spectrum blactamase, *MRSA=*methicillin-resistant *Staphylococcus aureus, J. thurifera=Juniperus thurifera*, MeE=Methanol extract, EtAE=Ethyl acetate extract, ChlE=Chloroform extract, PEE=Petroleum ether extract

The antibacterial activity of the organic extracts showed different inhibition profiles. But no activity of the four extracts was detected on all the Gram-negative tested strains. This could be due to the hydrophobic nature of the antimicrobial components present in the plant [18]. However, Gram-positive bacteria were inhibited (*S. aureus* ATCC and *S. aureus* MRSA), which match the results of *Juniperus drupacea* Labill. berries extract that showed no activity against Gram-negative tested bacteria [19]. Our results do not agree with the results of Sati and Joshi [20], who reported the inhibition of *J. communis* leaf extracts on Gram-negative and positive strains and also the results of Ennajar *et al*. [21] of the antibacterial activity of *Juniperus phoenicea* L. leaf extracts where the two groups of bacteria (Gram-positive and Gram-negative) showed their sensitivity, in addition to that it is found that the essential oil of *J*. *rigida* showed good antibacterial activity against the Gram-negative *Klebsiella pneumoniae*, with an inhibition zone diameter 16.00±0.25 mm and the lowest MIC and MBC values of 3.125 mg/mL [21]. On the other hand, essential oil of *J. excelsa* did not show any activity against *E. coli*, *P. aeruginosa*, or *S. aureus* [22].

**Figure-4:**
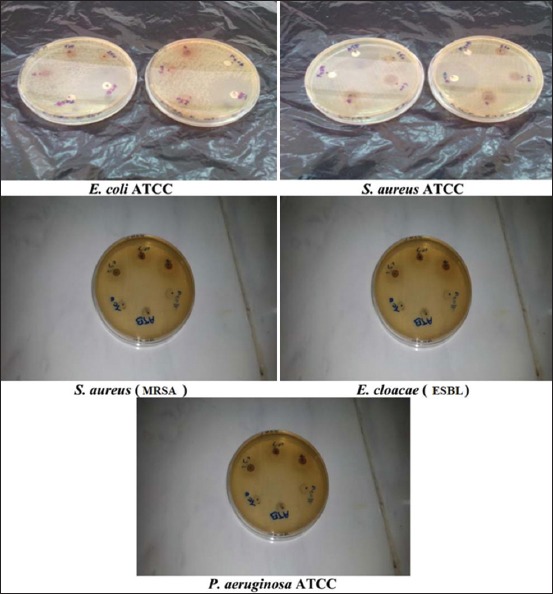
Antibacterial activity of *Juniperus thurifera* L. crude extract.

The MeE (Figure-4) exhibited the highest activity against *S. aureus* ATCC and *S. aureus* (MRSA) at the concentration of 1 g/mL with an inhibition diameter of 12 mm and 13 mm, respectively. However, the crude extract (methanolic) of *J. phoenicea* leaves showed high activity against *S. aureus* (15 mm inhibition zone at a concentration of 3 mg/mL) [20].

**Figure-5:**
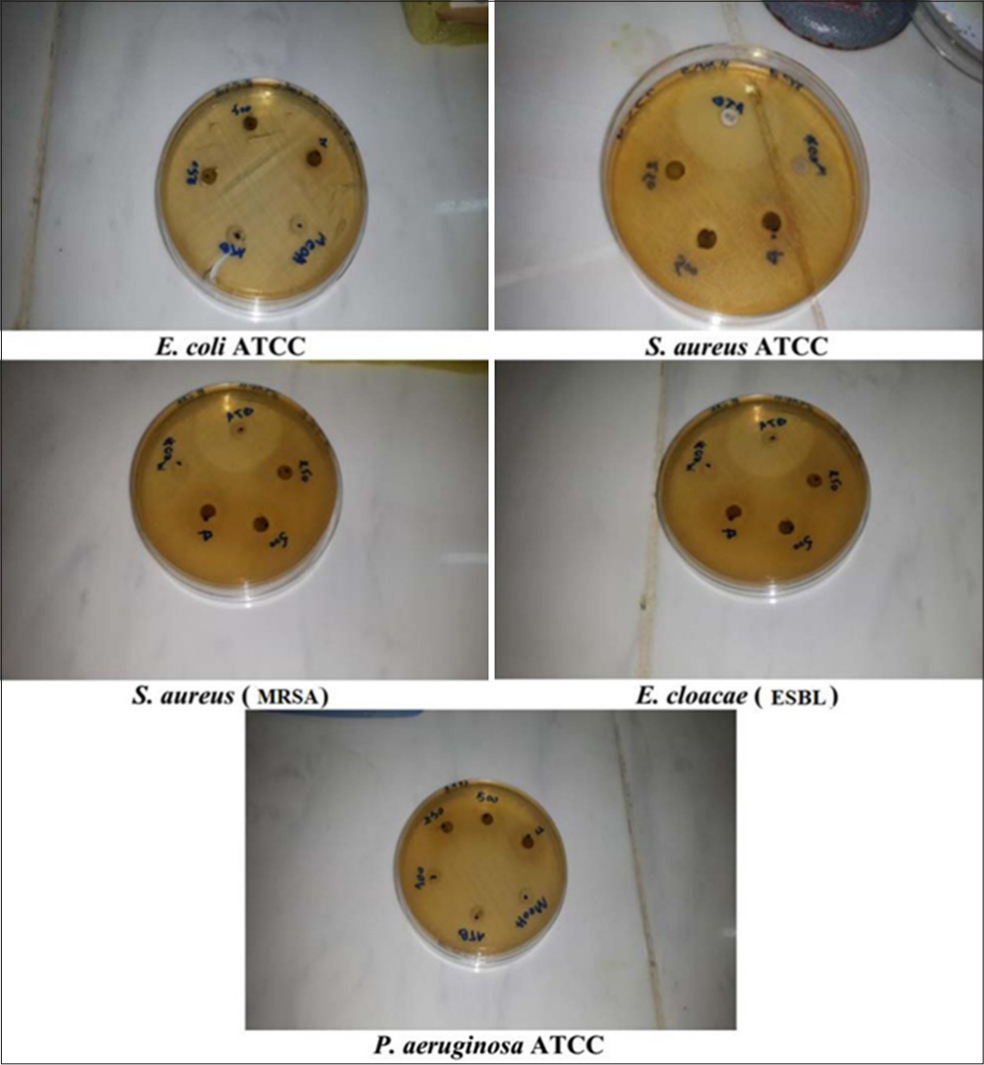
Antibacterial activity of *Juniperus thurifera* L. chloroform extract.

The ChlE (Figure-5) was the most effective against *S. aureus* MRSA among all the other extracts of the plant with a large diameter of 14 mm at the concentration of 1 g and a diameter of 11 mm against *S. aureus* ATCC for the same concentration.In contrast, the results of a study on the antibacterial activity of *J. excels*a leaf extracts [22] showed that the ChlE recorded an inhibition diameter of 8 mm at a concentration of 1 mg/mL against *S. aureu*s and against *E. coli* with a 9 mm inhibition diameter at the same concentration.

**Figure-6:**
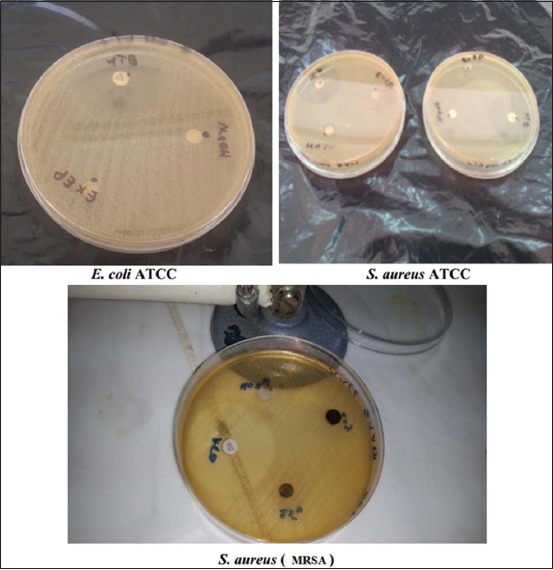
Antibacterial activity of *Juniperus thurifera* L. petroleum ether extract.

The PEE (Figure-6) had an inhibition diameter of 11 mm at 500 mg/mL for *S. aureus* ATCC and a smaller diameter at the same concentration (9 mm) against *S. aureus* MRSA.

**Figure-7:**
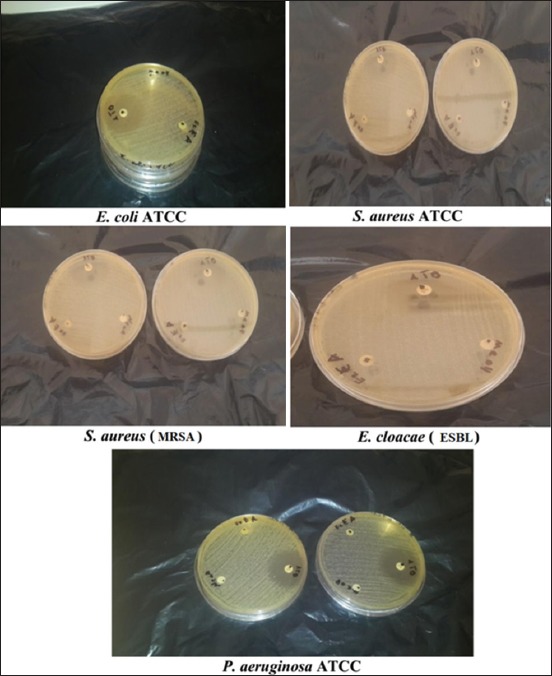
Antibacterial activity of *Juniperus thurifera* L. ethyl acetate extract.

The EtAE (Figure-7) did not show any activity against both Gram-positive and negative bacteria since the only tested concentration (100 mg/mL) was not enough.

The inhibitory activity of the organic extracts on *S. aureus* ATCC and *S. aureus* MRSA is very lower than the antibiotics.

From these results, it can be seen that whatever the nature of the extract and its concentration, Gram-negative bacteria possess strong resistance, which can be attributed to the difference in the structure of the cell wall.The cell wall of Gram-positive bacteria consists of a single layer and the absence of the outer membrane make these strains more vulnerable, while the Gram-negative bacterial cell wall has a multilayer structure with an outer cell membrane [23,24], containing phospholipids, proteins, and lipopolysaccharides and making this membrane impermeable to most biocidal agents [25,26].

## Conclusion

The present study has demonstrated that the leaf extracts of *J. thurifera* L. have considerable antioxidant activity and seem to be specifically effective against only Gram-negative bacteria including the clinical pathogen *S. aureus* MRSA. The above screening results enumerate the existing potential of plant chemical extracts to be used as a suitable candidate as medicine and pharmaceuticals and deserve to gain more interest to advance the research of the active molecules characterization present in this plant and their exploitation by the pharmaceutical and agro-alimentary industry.

## Authors’ Contributions

MM and AS designed the experiment protocol. MM, MR, MI, and AS carried out the experiment work. MM and HN were involved in data analysis and scientific discussion and drafted the paper; AA and OY revised the paper. All authors read and approved the final manuscript.
